# Supplementary material for: Effect of Liquefaction of Honey on the Content of Phenolic Compounds
Source: Molecules. 2023 Jan 11;28(2):714. doi: 10.3390/molecules28020714 (PMC9861181; doi:10.3390/molecules28020714)
Supplement: Supplementary file 1 [file molecules-28-00714-s001.zip › molecules-2113369-supplementary.pdf]

Effect of liquefaction of honey on the content of phenolic compounds

Tomáš Hájek*

Department of Analytical Chemistry, Faculty of Chemical Technology, University of Pardubice, Studentská 95, 532 10 Pardubice, Czech Republic

*Corresponding author, e-mail: tomas.hajek@upce.cz, tel.: +420 466 037 038

Supplementary materials

Figure S1. Effect of temperature on treatment time of honey in a microwave oven at a rated power of 90 W.

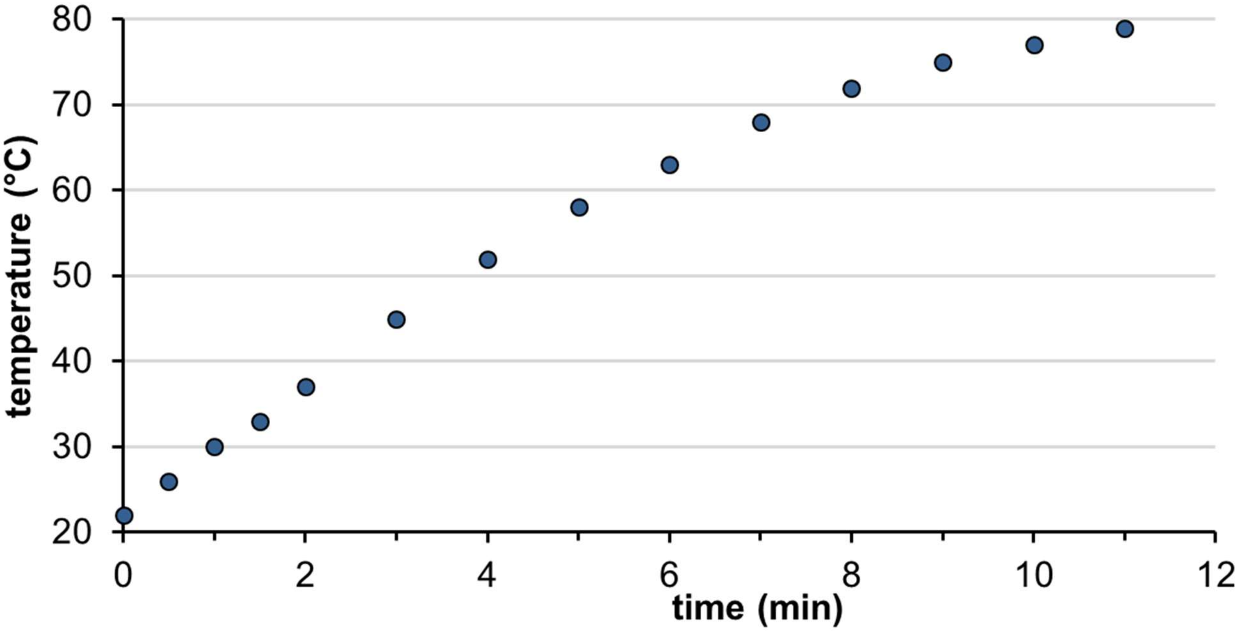

Table S1. Average concentration of tested compound in untreated honey and liquefied honey and standard deviation (SD, n=4). WB – water bath, USB – ultrasonic bath, MW – microwave.

	untreated honey		WB 60 °C		WB 80 °C		USB 60 W		MW 90 W		MW 270 W		MW 450 W		MW 900 W	
**Compound**	**conc.** (µg/kg)	**SD**	**conc.** (µg/kg)	**SD**	**conc.** (µg/kg)	**SD**	**conc.** (µg/kg)	**SD**	**conc.** (µg/kg)	**SD**	**conc.** (µg/kg)	**SD**	**conc.** (µg/kg)	**SD**	**conc.** (µg/kg)	**SD**
Protocatechuic a.	1461.1	4.2	565.2	28.8	537.1	15.8	431.0	18.4	482.7	23.8	552.9	22.8	564.7	15.1	404.0	7.5
Protocatechuic aldehyde	137.4	19.8	145.0	14.4	72.1	12.8	65.3	8.8	77.1	18.3	119.1	7.8	125.1	22.6	132.1	13.5
4-hydroxyphenylacetic a.	215.9	46.2	241.2	30.7	180.8	8.3	115.9	7.6	193.6	2.3	188.4	16.2	192.6	3.7	217.1	21.0
Chlorogenic a.	832.4	157.5	636.9	54.1	570.8	21.6	332.4	33.3	314.0	13.6	446.5	38.6	383.5	33.3	420.6	33.3
Rutin	204.3	44.3	92.6	10.1	91.5	4.3	98.4	21.3	62.3	1.8	122.1	15.3	107.1	13.0	130.0	9.4
Ethylvanillin	90.8	17.3	64.2	8.0	67.3	2.0	81.7	14.0	44.7	8.1	63.7	3.4	63.2	3.4	68.1	3.6

Table S2. Regression parameters of analyzed standards. R^2 - coefficient of determination.

No.	Compounds	Slope	Intercept	R^2
1	Gallic acid	9.00	0.64	0.9993
2	α-Resorcylic acid	10.75	6.00	0.9943
3	Protocatechic acid	10.14	0.08	0.9995
4	Gentisic acid	16.91	0.43	0.9990
5	Protocatechuic aldehyde	5.25	0.11	0.9991
6	β-Resorcylic acid	20.20	-2.46	0.9978
7	4-Hydroxyphenylacetic acid	21.61	-0.45	0.9988
8	4-Hydroxybenzoic acid	13.20	2.07	0.9954
9	Vanillic acid	14.16	2.44	0.9933
10	Chlorogenic acid	3.29	0.21	0.9980
11	Vanillin	13.13	0.88	0.9960
12	Isovanillin	12.05	-0.34	0.9958
13	*p*-Coumaric acid	2.55	-1.79	0.9903
14	Ferulic acid	2.94	-4.04	0.9986
15	Sinapic acid	4.95	-0.23	0.9987
16	Salicylic acid	5.95	-0.19	0.9952
17	Rutin	1.36	0.03	0.9918
18	Ethylvanilline	12.29	0.13	0.9992
19	Myricetin	3.48	0.68	0.9991
20	Quercetin	4.50	0.77	0.9903
